# Supplementary material for: Identification of Serum Interleukin-22 as Novel Biomarker in Pulmonary Hypertension: A Translational Study
Source: Int J Mol Sci. 2024 Apr 3;25(7):3985. doi: 10.3390/ijms25073985 (PMC11012889; doi:10.3390/ijms25073985)

Supplementary data

Figure 1. Correlation analyses of serum IL-22 levels with clinical parameters, using using Spearman’s rank correlation.

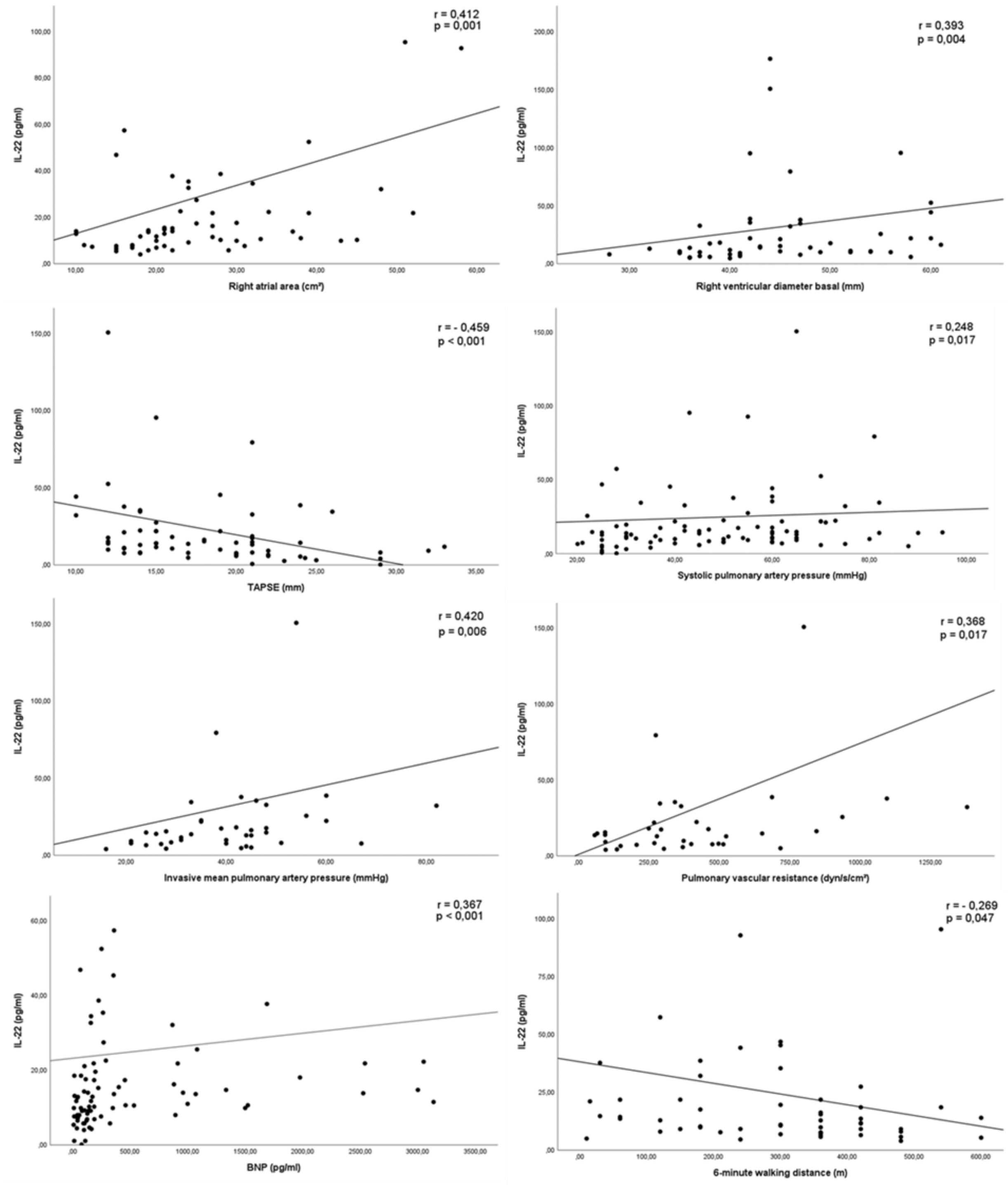

Figure 2. Serum IL-22 levels of patients in the functional class groups I - IV.

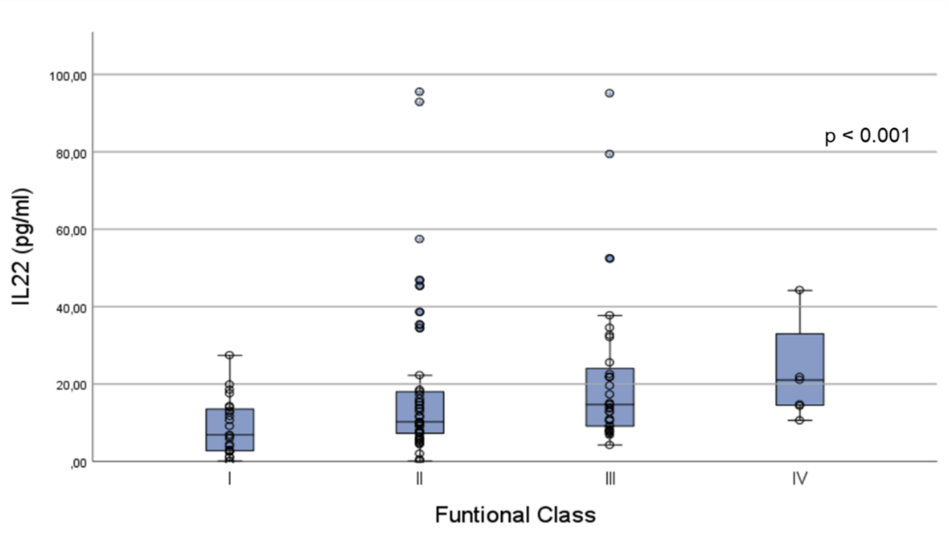

Supplement: Supplementary file 1 [file ijms-25-03985-s001.zip › ijms-2922671-supplementary.pdf]
